# Perceived coercion amongst healthcare workers during the COVID-19 pandemic

**DOI:** 10.1038/s41598-025-87700-6

**Published:** 2025-02-08

**Authors:** Andrea S. Stoltenberg, Veronica Ranieri, Hege Kristine N. Dahlen, Eleni Nastouli, SAFER Investigators, Matt Byott, Sarah J. L. Edwards, Daniel Frampton, Richard Gilson, Andrew Hayward, Jude Heaney, Catherine Houlihan, Fabiana Lorencatto, Ed Manley, Susan Michie, Moira Spyer, Nina Vora, Naomi Walker, Eleni Nastouli, Roger Almvik, Sarah J. L. Edwards, Sunjeev K. Kamboj

**Affiliations:** 1 Research Department of Clinical, Educational and Health Psychology, University College London, London, UK; 2 Great Ormond Street Hospital for Children, Psychological and Mental Health Services, London, UK; 3 Faculty of Medicine and Health Sciences, Norwegian University of Science and Technology, Trondheim, Norway; 4 Centre for Behaviour Change, Department of Clinical, Educational and Health Psychology, University College London, London, UK; 5 Department of Infection, Immunity and Inflammation, UCL Great Ormond Street Institute of Child Health, London, UK; 6 Centre for Research and Education in Security, Prison and Forensic Psychiatry, St.Olavs University Hospital, Trondheim, Norway; 7 Department of Science, Technology, Engineering and Public Policy, University College London, London, UK; 8 Department of Clinical Virology, University College London Hospitals NHS Trust, London, UK; 9 Department of Infection and Immunity, University College London, London, UK; 10 Institute for Global Health, University College London, London, UK; 11 Institute of Epidemiology and Healthcare, University College London, London, UK; 12 School of Geography, University of Leeds, Leeds, UK; 13 Department of Experimental Medicine, Liverpool School of Tropical Medicine, Liverpool, UK

**Keywords:** Perceived coercion, Scale validation, Pandemic, Healthcare workers, Psychological distress, Psychology, Health care

## Abstract

Direct and prolonged exposure to stress and uncertainty among healthcare workers (HCWs) during the COVID-19 pandemic likely had a significant negative impact on their mental health and general wellbeing. Although the contributors to such distress remain to be fully understood, the construct of *perceived coercion* appears to be relevant. Among HCWs, perceived coercion is conceptualised as appraisals about lack of control/‘freedom to choose’ and pressure to perform patient-care duties in the context of unprecedented threat of contagion from patients. To improve our understanding of perceived coercion amongst HCWs, we developed a 10-item scale—the Pandemic-specific Perceived Coercion Scale for Healthcare Workers (PPCS-HCW) scale—designed to be adaptable and applicable for use in future mass-contagion emergencies. A preliminary (exploratory) factor analysis (*N* = 546) showed that relevant items coalesced around three factors—‘internal pressure’, ‘external pressure’ and ‘perceived coercion’, that partly overlap with previous conceptualisations of perceived coercion. The exploratory conceptual and psychometric structure was confirmed in a separate sample of HCWs from the UK and Norway (*N* = 483). *On average*, across the three PPCS-HCW scale factors, HCWs showed low levels of perceived coercion (*M* = 0.22 (95% CI [0.11, 0.33] on a − 3 to + 3 scale). However, cluster analysis identified three groups: low (− 1.09 (95% CI [− 1.20, − 0.99]), moderate (0.17 (95% CI [0.08, 0.25]) and high scoring (1.57 (95% CI [1.47, 1.67]) PPCS-HCW clusters. High scoring participants showed higher levels of psychological distress, avoidance coping and compassion fatigue. In summary, our findings suggest that perceived coercion is a relevant construct in understanding the adverse psychological impact of large-scale contagion emergencies on HCWs.

## Introduction

The COVID-19 pandemic led to unprecedented challenges for healthcare workers (HCWs) across the globe^1,2^ In fulfilling their frontline roles, HCWs were expected to manage stressful and unfamiliar work conditions, high levels of uncertainty and exposure to an infectious and potentially fatal disease caused by a novel virus (e.g.,^3–5^). In sharp contrast to the contact restrictions placed on the general population, HCWs were *expected* to put themselves at a higher risk of infection in order to perform their professional duties and protect the general population from harm^6^. The risks of harm were particularly salient in the context of shortages of healthcare resources and limited personal protective equipment (PPE). These conditions resulted in difficult ethical and moral professional dilemmas^7^. Numerous studies have investigated COVID-19-related stressors contributing to elevated rates of psychological distress in HCWs during previous large-scale health emergencies, as well as the COVID-19 pandemic e.g.^3,8,9^. Rather than merely being a result of excessive workloads and exposure to risk, factors such as lack of strict and consistent infection control guidelines, PPE, collegial support, media coverage, stigmatization, isolation, lack of access to structured mental health care, and fear of infecting others, have all been associated with negative psychological impact^3,10–18^. Healthcare staff were also at increased risk of moral injury, the psychological distress that results from actions or inaction which violate someone’s moral code or perceived professional standards as set out in case law, further contributing to anxiety, depression, PTSD symptoms and alcohol misuse in HCWs during the pandemic^5,19^.

Although the adverse impact of the COVID-19 pandemic on HCWs’ mental health is widely acknowledged, less is known about how the professional dilemmas HCWs face during mass contagion emergencies, when their bodily and psychic selves were at risk *as a result of* performing their professional duties, impacted their mental health. A concept that might help us develop our understanding of vulnerability to psychological distress amongst HCWs under these circumstances is *perceived coercion*. Perceived coercion has been widely studied in the context of detention of psychiatric patients requiring mental healthcare and refers to an individual’s appraisal of being coerced or pressured to do something and believing that refusing to comply will lead to negative or harmful consequences for them^20^. Perceptions of coercion may arise from feeling excluded from decision-making, feeling that a situation is forced upon one without justification, or feeling unable to express one’s true opinions. It has been recognised that sources of perceived coercion and pressures can be internal and external and are predominately ‘informal’ influences, such as internal and social pressures and norms, rather than formal or legal factors^20–22^. Perceived coercion may, therefore, arise in the absence of external force/pressure, and similarly, external force may not be experienced as coercive. As such, individuals might experience (i.e. perceive) different ‘types’ and intensities of coercion, especially in circumstances where their needs and preferences are subordinate to a larger system within which they lack agency. High levels of perceived coercion are detrimental to wellbeing e.g.,^23,24^ *c.f.*^25^. For instance, in psychiatric patients, perceived coercion is associated with post-traumatic stress symptoms^26^, lower quality of life, and impaired psychosocial functioning^27^. However, the concept of perceived coercion appears to have broader application beyond psychiatric care.

Building on Szmukler and Appelbaum’s (2008) definition of perceived coercion and applying it to individuals within healthcare systems during the COVID-19 pandemic, HCWs facing stressful and complex professional and personal decision-making might have experienced significant self-discrepancy and distress in the context of their professional caring role. On one hand, they had a professional obligation and duty of care to their patients; on the other, they may have felt fearful of infection and unable (due to resource limitations) to adequately provide this care^7,28,29^. Relevant ‘external pressures’ faced by HCWs might have related to concerns that failure to comply with the expectations to heroically work on the frontline^30^ would result in unfavourable evaluations from their employers and/or the public. Internal pressures might have included internalised professional standards and ideals. Simultaneously, and potentially in (perceived) conflict with these pressures, self-preservation was likely to have been a strong motivational driver for HCW during the most severe periods of the pandemic. HCWs were therefore faced with two options, both of which had negative consequences for them; (i) putting themselves, their patients and loved ones at risk of infection (especially if PPE was inadequate) whilst accepting further compulsory conditions (e.g., vaccination, unfamiliar work responsibilities, longer working hours), or (ii) refusing to work with COVID patients, risking professional sanctions and stigmatization. Violations of the internal standards (i.e., the values that often lead HCWs to the caring professions in the first place; e.g. own-ought: actual-self discrepancy^31^, professional expectation to subordinate their needs to their patients’, and media portrayals of HCWs as heroes^30^ may have contributed to perceptions of pressure, or coercion, to accept risks of infection while caring for patients^7^.

Investigating perceived coercion in HCWs therefore seems crucial to developing a better understanding of the contributors to poor mental health among HCWs during the COVID-19 pandemic e.g.,^3^. This could inform responses to future pandemics or other large-scale health emergencies in which HCW are asked to put themselves at risk in the interest of their patients and the public. This requires the development of reliable instruments for assessment. Although a number of existing scales have been widely used in mental health research to measure the impact of a range of restrictive practices, the intended respondents are patients rather than professionals delivering care. Though the emphasis differs between these scales in their definition of perceived coercion, they all tend to recognise core components of the construct, namely perceived coercion per se (e.g., lack of control and freedom) and perceived pressures (i.e., the perception that forces are acting on the individual to drive their behaviour in a manner that conflicts with their wishes). Moreover, the multifaceted nature of perceived coercion requires a multi-factor measurement instrument that meaningfully captures a range of personal, and context-specific aspects of perceived coercion^32^. Given the absence of a relevant measure of perceived coercion among healthcare workers, we drew on existing scales to develop a new instrument during the COVID pandemic: the Pandemic-specific Perceived Coercion Scale for Healthcare Workers (PPCS-HCW), evaluated its psychometric characteristics and tested the relationship between perceived coercion and distress among HCWs. Preliminarily, we also examined whether perceived coercion was associated with compassionate fatigue and coping styles (avoidant and approach coping) given that these are associated with distress and occupational impairment in HCWs e.g.^33,34^ and could contribute to an evaluation of convergent/discriminant validity of the PPCS-HCW. Although the development and evaluation of the PPCS-HCW occurred during the COVID-19 pandemic, we aimed to develop an adaptable measure that could be modified for use in research on pandemic-related perceived coercion and distress/impairment in HCWs in future global contagion emergencies.

## Materials and method

The study received ethical approval from the University College London Research Ethics Committee and all procedures were carried out in line with the principles outlined in the Declaration of Helsinki on research involving human subjects. Questionnaire development was undertaken in three phases, as outlined by Boateng et al.^35^. These occurred during periods of active infection threat in a relatively early period of the pandemic (see below). In Phase 1, we generated an item pool using a combination of deductive methods, via a review of the existing literature on perceived coercion (scales), and inductive methods, using semi-structured interviews with healthcare workers. Face and content validity of preliminarily generated items were tested in a separate group of HCWs (different to those involved in the inductive process of initial item development). Phases 2 and 3 consisted of two cross-sectional studies with frontline healthcare workers from inpatient and outpatient settings and were intended to explore (Phase 2) and then confirm (Phase 3) the factor structure of the instrument in healthcare workers in the UK (Phase 2 and 3) and Norway (Phase 3) and also to explore the relationship between the underlying construct of perceived coercion and relevant psychological variables. All data for these phases was obtained via online surveys hosted on Qualtrics^36^ and Opinio (Object Planet^37^).

### Participants

Eligible healthcare professionals were clinicians aged ≥ 18 years who had experience working on the frontline of the pandemic, defined in the advertisement as having worked directly with or in the same environment as confirmed or possible COVID-19 patients. Convenience sampling and snowballing was employed to recruit the required large sample. Biases associated with such a sampling strategy (e.g., the representativeness of the sample), had to be balanced against resource limitations and the time-sensitive nature of the scale development task.

In Phase 1, n = 20 HCWs were recruited to develop and refine items. An initial n = 10 doctors and nurses were interviewed to understand the nature of coercion, to adapt and add items, and to understand which aspects of coercion were most important to assess in the context of the COVID-19 pandemic. A separate mixed group of HCWs with frontline duties during the pandemic (n = 10) piloted the initially identified items for face and content validity.

Phase 2 participants (the ‘exploratory sample’) were a cross-sectional sample of nurses, doctors and other frontline hospital staff (n = 546) in the UK. Due to resource constraints, recruitment for phase 2 occurred in two waves between July and October 2020 (highest average number of SARS-CoV-2 cases ~ 20 000) and January and May 2021 (highest average number of SARS-CoV-2 cases ~ 60,000). To examine perceived coercion in a wide range of HCWs from different sectors, the study was advertised through (i) social media (Twitter, Facebook, Instagram and WhatsApp) and (ii) emailed to HCWs via cooperating organisations in the UK National Health Service (NHS) and other universities and colleges involved in training NHS staff, and (iii) emailed to HCWs who had consented to be contacted as part of a larger study (SARS- CoV-2 Acquisition in Frontline Healthcare Workers; SAFER^38^. Although convenience sampling was used, we attempted to encourage diversity among participants by advertising the study through organisations and social media groups that represented professionals from different ethnic backgrounds. The advert or email to participants contained a link which took participants to the study landing page with study information and a consent form. After confirming they had read the study information and were willing to proceed with the study, participants provided informed consent, recorded electronically, before proceeding to the main survey, which included the preliminary items of the PPCS-HCW. The survey was discontinued if participants indicated that they had not worked with confirmed or suspected COVID-19 patients. The PPCS-HCW was included as part of larger online surveys consisting of demographic details and background information, questionnaires measuring psychological distress and coping styles, as well as behaviour change measures to reduce transmission rates.

Phase 3 participants (n = 483; the ‘confirmatory sample’) were recruited using a similar strategy to phase 2, although participants included HCWs from both the UK and Norway. UK data collection occurred between mid-November 2020 and early April 2021(highest average number of SARS-CoV-2 cases ~ 60,000) and Norwegian data collection occurred between early January 2021 to early April 2021 (highest average number of SARS-CoV-2 cases ~ 25,000). Policies that were in place for the general population during these time periods included school closure/remote education, restricted social contact, encouragement to work from home, mask requirements and testing and quarantine in relation to travel and suspected contact with SARS-CoV-2 cases.

The number of participants recruited to Phases 2 and 3 was not pre-determined through a sample size calculation. Rather, we sought to recruit as many participants as possible within the relevant limited timeframe to enable us to perform the psychometric validation procedures using accepted rule-of-thumb sample sizes (i.e., n > 300).

### Item development

As noted above, the initial set of items for the Pandemic-specific Perceived Coercion Scale for Healthcare Workers (PPCS-HCW) were formulated by reviewing existing perceived coercion scales and interviewing healthcare workers with experience of working on the frontline at early stages of the pandemic. Most items were initially adapted from the MacArthur Admission Experience Survey^39^ and other perceived coercion scales (e.g., Perceived Coercion Questionnaire, Coercion Experience Scale, the Visual Analogue Coercion Ladder scale). In line with these scales, the PPCS-HCW was intended to assess the two distinct aspects of perceived coercion namely, perceived coercion per se and perceived pressures, including internal pressure (i.e., internally generated standards and beliefs) and external pressure (e.g., professional, public, management, peer pressures). Bilingual translation and backtranslation were used^40^ and cross checked with Norwegian and English members of the research team. The initial 15 items were piloted and modified in response to interviews and feedback. In particular, two items adapted from the McArthur Admission Experience Survey^39^ were dropped (“I chose to work with COVID-19 patients” and “I was willing to work with COVID-19 patients”) because the majority of HCWs had not been offered a choice in working with COVID-19 patients. The preliminary 13 item PPCS-HCW included instructions asking participants to indicate how strongly they agreed or disagreed with a number of statements, ranging from 1 (*strongly disagree*) to 7 (*strongly agree*). Note, this scaling was modified (see below) to − 3 to + 3 to make interpretation easier. The final (10-item) English and Norwegian version of the scale is presented in the Supplement.

### Psychometric evaluation

Although there is the general three-factor framework for perceived coercion discussed above, it was uncertain whether the factor structure of the PPCS-HCW would conform to this model. We therefore examined the factor structure of the PPCS-HCW and validated the measure using the common two-step exploratory-confirmatory procedure. Exploratory factor analysis (EFA) and internal consistency were conducted using SPSS (Version 27). Confirmatory factor analysis (CFA) was performed within a structural equation modelling framework using AMOS (Version 26.0).

The suitability of the data for EFA was based on the sample size, and an investigation of the factorability of the correlation matrix and the Kaiser–Mayer–Olkin Measure of Sampling Adequacy. Principal axis extraction and oblique rotation^41^ were used to allow extracted factors to correlate. A variety of criteria were considered in deciding the number of factors to retain: (scree plot of) eigenvalues, parallel analysis (adapted from Patil et al.,^42^), total variance accounted for by retained factors, inter-factor correlations, and item-level factor loadings^43^.

The items within each putative factor were also investigated for cross-loadings, internal consistency, and the presence of a common theme with a coherent interpretative and theoretical basis. In an effort to obtain factors with simple structures (i.e., containing items that loaded largely on a single factor), items with cross-loadings were considered for exclusion. However, the decision to exclude was balanced against considerations of interpretative, theoretical, and practical value. Factors that consisted of three or more items with salient pattern coefficients ≥ 0.40 were considered adequate for inclusion as distinct constructs^41,44^. Factors with an internal consistency > 0.70 were considered adequate^45^.

Based on the constraints (factors structure) implied by the EFA, a CFA was performed on data from a separate sample of participants from the UK and Norway. Measurement invariance across the UK and Norwegian sample was assessed using multi-group CFA. As suggested by^46^, three steps were used: configural (i.e., equivalence of model organisation/form), metric (i.e., equivalent of factor loading) and scalar invariance (i.e., equivalence of item intercepts). The fit of metric, scalar and residual invariance models were evaluated by computing the difference between fit statistics for two nested models that were identical except for a target set of restrictions in one. Invariance across the three steps indicates that an instrument can meaningfully be used across contexts (countries in this case). If measurement invariance is achieved, a CFA of the combined dataset is appropriate to confirm the factor-structure with a larger combined sample.

Model fit was tested through chi-square (χ^2^), the Comparative Fit Index (CFI), Tucker Lewis Index (TLI) and Root Mean Square Error of Approximation (RMSEA). Values ≥ 0.95 for CFI and TLI, and < 0.05 on the RMSEA are widely considered to be indicative of close-fitting models. However, CFI and TFI values > 0.90^47^ and RMSEA values up to 0.10 are considered acceptable^48^. Measurement invariance indices were assessed with alternative fit indices (AFIs) including chi-square (χ^2^), Root Mean Square Error of Approximation (RMSEA), Comparative Fit Index (CFI), and McDonald’s^49^ Noncentrality Index (McNCI), with the following criterion values: Δχ^2^, *p* > 0.05, ΔRMSEA < 0.01, ΔCFI < 0.01, ΔMcNCI < 0.02.

### Descriptive statistics and scoring

To aid interpretability of the PPCS-HCW, the seven response options anchored between “strongly agree” and “strongly disagree” (inclusive) were assigned values between + 3 and − 3 respectively, with a neutral “neither agree/disagree” = 0 anchor in the middle. Higher (more positive) values indicated greater perceived coercion.

This scoring scheme was applied to the phase 3 survey results. A k-means cluster analysis was performed using standardized subscale scores from the phase 3 sample. A three-cluster solution was specified, to allow participants to be classified as belonging to low, moderate or high perceived coercion groups. Univariate ANOVA was used to compare these three groups on key continuous variables whereas χ^2^ goodness of fit tests were used to evaluate the distribution of participant characteristics (sex, country of residence, professional role, etc.) in each of the three groupings. Where control variables were included in the model, univariate ANCOVA was used.

### Other measures

As part of a larger programme of research a number of other measures were taken as part of the survey^50^. These included demographic and workplace characteristics and history of health (including mental health) problems. Of relevance to the current paper, mood was assessed using the Depression Anxiety and Stress Scale (DASS; 21 item version: DASS-21:^51^. The DASS-21 consists of three subscales (depression, anxiety and generalised stress), each consisting of seven items (scored 0–3). The total score is multiplied by two to allow comparison with the common, longer version of the scale. Additionally, a single total score on compassion fatigue was determine from the ProQOL-21^52^. As recommended by Carver and colleagues, items from the Coping Orientation to Problems Experienced Inventory (28-item Brief-COPE:^53^ were factor analysed to identify avoidant and approach items (10 and 12 items respectively). Items tapping substance use, humour and religion^54^ were excluded for psychometric and conceptual (low relevance to the sample: substance use) reasons.

Participants also completed a single-item measure of how well supported they felt by their team: “I felt supported by my team”. This was measured on a 1–7 scale (1 = not at all; 7 = very much).

### Overlapping content

An earlier version of this paper was published on the pre-print service: PsyArXiv (2023): 10.31234/osf.io/j897b.

## Results

### Descriptive statistics

For the exploratory (EFA) sample (*N* = 546) demographic and occupation characteristics are presented in Table 1. The age of the sample ranged widely (18–72 years), with a mean of 41 (SD = 11.4) years. A range of frontline healthcare professional roles were represented, including nurses, medical doctors, allied health professionals, midwifes, and pharmacists. Despite attempts to encourage participation across ethics groups, the final sample was predominantly white.

**Table 1:** Participant characteristics in exploratory factor analysis sample (N = 546).

Participant characteristics	N (%)
UK country of residence, England	522 (95.6)
Gender*, Female	430 (81.3)
Ethnicity*, Caucasian/White	421 (79.6)
Role*	
Nurse	187 (35.3)
Medical Doctor	118 (22.3)
Other	225 (42.4)
Workplace*	
Accident & Emergency	44 (8.4)
Intensive Care	31 (5.9)
COVID-19 ward	16 (3.0)
Other	435 (82.8)
Redeployment*, Yes	51 (9.6)
Diagnosed COVID-19, Yes	164 (31.1)

* = variable with missing data (< 4% of cases missing). Other (role) = allied health professionals (dieticians, occupational therapists, physiotherapists, paramedics etc.), midwife, pharmacist, and other care workers with direct patient contact. Other (workplace) = inpatient and outpatient settings e.g., acute medical ward, haematology ward, obstetrics & gynaecology ward, virology ward, surgical ward, primary care settings, prehospital setting, hospice, and ambulance.

### Item selection and factor structure

Thirteen participants were excluded listwise due to one or more missing values for individual items, leaving a total of n = 534 included in the phase 1 EFA. Of the 13 items of the PPCS-HCW that were based on previous measures of perceived coercion and HCW interviews and feedback, two items (“I had more influence than other health professionals on deciding whether I…”; “I did not feel professionally obliged to…”) were excluded on the basis of low correlations with other items, poor communalities (< 0.40) and/or poor factor loadings (< 0.40). As such, the resulting scale consisted of 11 items.

The correlation matrix of scores for the 11 items indicated sampling adequacy (Keiser-Meyer-Olkin statistic = 0.86;^55^ and hence, appropriateness for factor analysis. A consideration of the various criteria for establishing the number of factors to retain (especially parallel analysis; see Methods) suggested a 3-factor solution for the PPCS-HCW, which accounted for 55.8% of item variance.

Table 2 shows the factor loadings for each item within its appropriate factor after rotation. The items that loaded on the first factor generally represent internal and informal pressures related to the healthcare worker role, including concerns about deviating from professional expectations and internal standards (Internal Perceived Pressure; IPP). The second factor represented general experiences of coercion especially relating to (lack of) control and autonomy and expectations of professional peers (Perceived Coercion per se; PC). The third factor represented the experience of compulsion from external sources and consequences of non-compliance (External Perceived Pressure; EPP).

**Table 2:** Factor loadings and factor correlations for oblimin rotated three-factor solution for PPCS-HCW.

Item	Rotated factor loading		
	1	2	3
Internal Perceived Pressure (IPP)			
I worried about not living up to my profession if I refused to work with patients with COVID-19	0.64	–	–
I worried about the potential burden on my colleagues if I refused to work with patients with COVID-19	0.68	–	–
I worried about what others would think of me if I refused to work with patients with COVID-19	0.60	–	–
**The public expected me to work with patients with COVID-19 in spite of the risk (removed)**	**0.49**	–	–
Perceived Coercion subscale (PC)			
I had a lot of control over whether I worked with patients with COVID-19	–	0.72	–
If I wished to, I could have refused to work with patients with COVID-19	–	0.67	–
My peers expected me to work with patients with COVID-19 in spite of the risk	–	0.58	–
Superiors expected me to work with patients with COVID-19 in spite of the risk	–	0.56	–
External Perceived Pressures (EPP)			
Somebody forced me to work with patients with COVID-19	–	–	− 0.82
Somebody pressured me to work with patients with COVID-19	–	–	− 0.75
I worried about the potential personal consequences of refusing to work with patients with COVID-19*	–	–	− 0.45
Cumulative variance (%)	40.6	48.7	55.8
Cronbach’s alpha (α)	0.76	0.79	0.82

	Factor correlations		
	1	2	3
1	1.00		
2	0.40	1.00	
3	− 0.38	− 0.38	1.00

*indicates that the item remained cross-loaded with different rotation methods (i.e., Promax, Varimax). The factor in which they were eventually retained was dictated by a higher loading for that factor and/or conceptual similarity with other items within the factor. The item in bold (The public expected me…..) was removed in the CFA described below.

The modest inter-factor correlations suggest independence of the subscales and each subscale’s Cronbach’s alpha was ≥ 0.75, suggests adequate internal consistency (see Table 2). In sum, consistent with the literature on perceived coercion, the analysis revealed three underlying factors in the PPCS-HCW that appear to correspond to previously described sub-components of perceived coercion, namely perceived coercion per se, and internal and external sources of perceived pressure.

### Confirmatory factor analysis

Participants included in the CFA from the two countries had similar characteristics. The pooled demographic and professional characteristics are described Table 3.

**Table 3:** Participant characteristics (N = 483) of the confirmatory factor analysis sample.

	Mean (SD)
Demographics	
Country of residence, n UK (%)	197 (40.8)
Age, years	40.25 (11.23)
Female, n (%)	334 (69.2)
Caucasian, n (%)	434 (89.9)
Married/in a relationship, n (%)	339 (70.2)
Postgraduate degree, n (%)	243 (50.3)
Annual household income, n > £50,000/yr (%)	367 (76.1)
Workplace factors	
Role	
Nurse, n (%)	181 (37.5)
Doctor, n (%)	190 (39.3)
Other, n (%)	112 (23.2)
Workplace	
Accident and Emergency, n (%)	57 (11.8)
Intensive care, n (%)	147 (30.4)
COVID-19 ward, n (%)	47 (9.7)
Other, n (%)	361 (74.7)
Years of Experience, years	15.35 (10.56)
Redeployment, yes (%)	119 (24.6)
Perceived Coercion Subscales	
Internal Perceived Pressure^a^	0.40 (1.59)
Perceived Coercion (subscale)^a^	1.10 (1.31)
External Perceived Pressure^a^	− 0.84 (1.65)
Perceived Coercion Total^a^	0.22 (1.22)
Psychological Distress	
Stress	11.96 (10.35)
Anxiety	7.07 (8.19)
Depression	10.97 (10.85)

All data provided as mean (SD) unless indicated otherwise. SD = standard deviation. Participants could choose several workplaces, which is reflected in the n (%) above. Psychological distress based on the Depression, Anxiety and Stress Scale (DASS-21): Depression score range in the sample: 0–42, Anxiety score range: 0–38, Stress score range: 0–42. Note, the values on the DASS-21 were derived by multiplying the raw scores by two, as recommended, to allow for comparison with the long-form DASS. ^a^To facilitate interpretation the average scores based on the 1–7 range were rescaled from − 3 to + 3, such that − 3 = strongly disagree, 0 = neither agree nor disagree and + 3 = strongly agree.

Internal consistency of the PPCS-HCW, collapsed across countries, for the perceived coercion subscale (4 items, α = 0.72) and external perceived pressure (3 items, α = 0.80) subscales was adequate. However, reliability of the internal perceived pressures subscale was questionable (α = 0.68), with one item (i.e., ‘the public expected me to…’) demonstrating poor item-total correlation (*r* = 0.23) and seeming to contribute to the lower reliability score. Dropping the item led to acceptable reliability for this subscale (3 items, α = 0.74). The item was excluded based on these psychometric considerations.

**Fig. 1:**
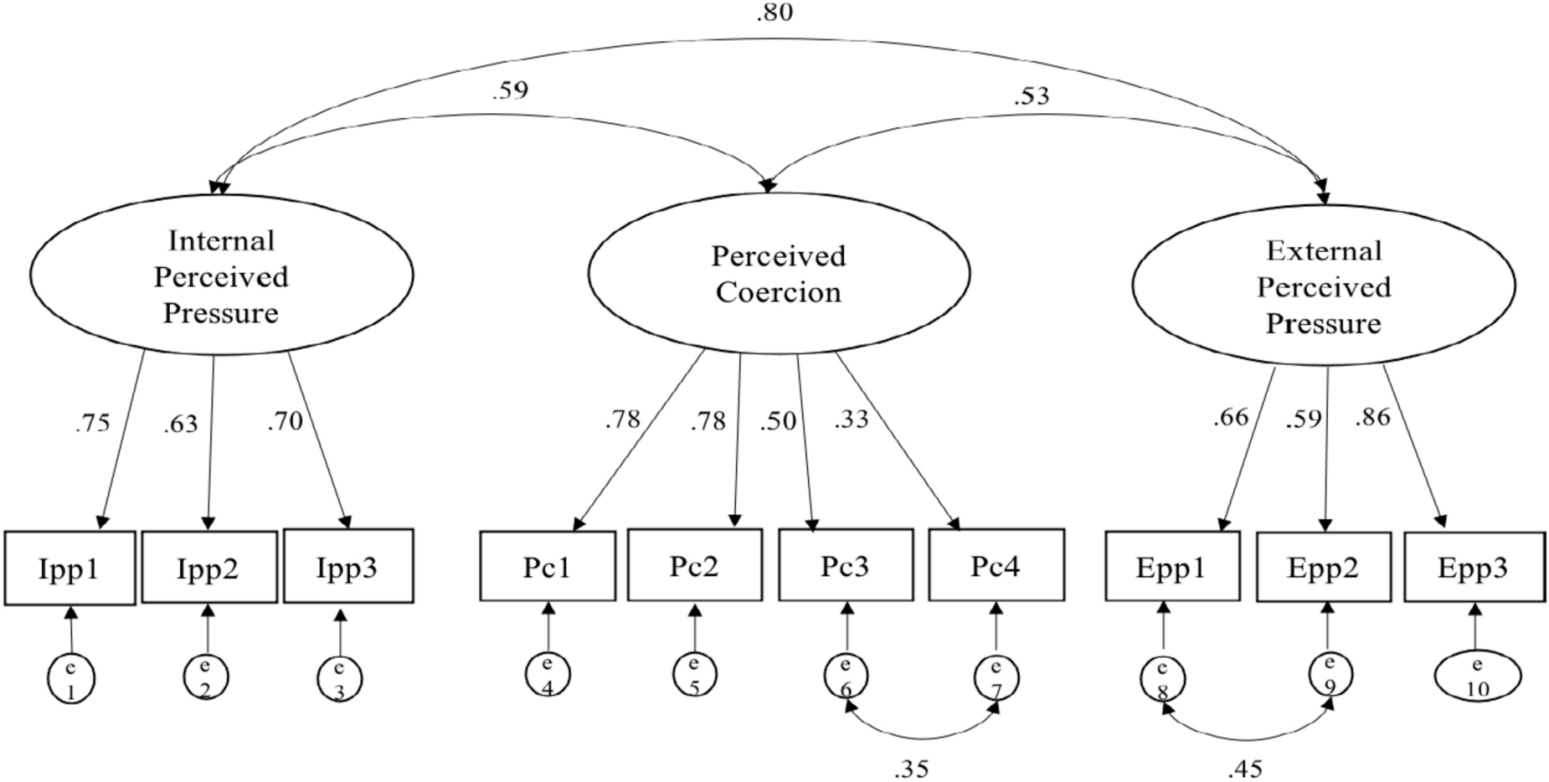
Confirmatory factor analysis with the combined dataset (N = 483). *Note*: Ipp = Internal perceived pressure items; Pc = perceived coercion subscale items; Epp: external perceived pressure items. Ipp1 = I worried about not living up to my profession if I refused, Ipp2 = I worried about the potential burden on my colleagues if I refused, Ipp3 = I worried about what others would think of me if I refused, Pc1 = My peers expected me to work with patients with COVID-19 in spite of the risk, Pc2 = Superiors expected me to work with patients with COVID-19 in spite of the risk, Pc3 = I had a lot of control over whether I worked with patients with COVID-19, Pc4 = If I wished to, I could have refused to work with patients with COVID-19, Epp1 = Somebody pressured me to work with patients with COVID-19, Epp2 = Somebody forced me to work with patients with COVID-19, Epp3 = I worried about the potential personal consequences of refusing. Allowing the error terms between Pc3 and Pc4 and between Epp1 and Epp2 to be correlated was justified by method effects (reverse scored items or closely related wording respectively).

A multi-group confirmatory factor analysis was performed on the 10-item version of the PPCS-HCW to test for measurement invariance between countries. Modification indices suggested methods effects between two perceived coercion (subscale) items that were reverse coded (i.e., Pc3 and Pc4) and two that were similar in wording/meaning from the external perceived pressure subscale (i.e., Epp1 and Epp2) (see Fig. 1). The error terms for these items were therefore allowed to covary.

To confirm measurement invariance between countries, configural, metric and scalar invariance were tested. The χ^2^ value (χ^2^ (60) = 197.81, *p* < 0.001) for the configural (baseline) model indicated potentially poor model fit, and so other fit indices were examined. On balance, the values obtained—CFI = 0.92; TLI = 0.88; RMSEA = 0.07—confirmed the 3-factor solution in both countries and configural invariance. Metric invariance was achieved, however full scalar invariance was not supported. Full measurement invariance is rarely supported and so it is common practice to accept some violation of measurement invariance by releasing constraints on one or more factor loading(s) or intercept(s)^46^. Partial scalar invariance was established with a minimum of two intercepts restricted in each factor^46^. Thus, as can be seen in Table 4, measurement invariance was achieved.

**Table 4:** Measurement invariance analysis between countries (UK n = 197, Norway n = 286).

Model	χ^2^(*df*)	CFI	RMSEA	McNCI	MC	Δχ^2^Δ*df*)	ΔCFI	ΔRMSEA	ΔMcNCI	Decision
M1: Configural invar	197.8 (60)	0.92	0.069	0.87	–	–	–	–	–	Accept
M2: Metric invar	207.6 (67)	0.92	0.066	0.87	M1	9.75 (7)	0.001	0.003	0.002	Accept
M3: Scalar invar	264.8 (74)	0.89	0.073	0.82	M2	57.26 (7)*	0.029	0.007	0.044	Reject
M4 Partial scalar invar	213.4 (70)	0.92	0.065	0.86	M3	5.85 (3)	0.002	0.001	0.003	Accept

* < 0.001, MC = Model Comparison. Thresholds for measurement invariance: Δ chi-square *p* > 0.05, ΔRMSEA < 0.01, ΔCFI < 0.01, Δ Mc NCI < 0.02.

Given the invariance described above, the datasets from the two countries were combined to confirm the 3-factor-solution. On balance, though not a close-fitting model, the values of the indices in the combined CFRA indicated acceptable fit, CFA, χ^2^ value (χ^2^ (30) = 143.77, *p* < 0.001), CFI = 0.93; TLI = 0.90; RMSEA = 0.09 [CI 0.07, 0.10], confirming the 3-factor solution. Factor loadings and correlation indices can be found in Fig. 1.

### Preliminary description of perceived coercion among healthcare workers during the COVID-19 pandemic

Based on the likely factor structure of the PPCS-HCW, we provide a preliminary description of perceived coercion, internal pressure and external pressure among HCW in the UK and Norway in Table 3.

On average, participants scored reliably greater than 0 on the internal perceived pressure (*M* = 0.40 (95% CI [0.26, 0.55]) and the perceived coercion subscales (*M* = 1.10 (95% CI [0.98, 1.22]). However, they scored reliably *less than 0* on external perceived pressure, generally indicating a tendency for an absence of perceived external pressure (*M* = -0.84 (95% CI [− 0.99, − 0.70]). Averaged across the three factors, perceived coercion averaged across the 10 items was marginally (though reliably) greater than 0 (*M* = 0.22 (95% CI [0.11, 0.33]). Taken together, perceived coercion was low amongst our sample of HCWs, although the dispersion in scores suggested the potential for different groups of participants with differing levels of perceived coercion.

#### Demographic characteristics of healthcare workers with low, moderate and high perceived coercion

The ordered means for low, moderate and high PPCS-HCW subscales obtained from the k-means cluster analysis are summarised in Table 5, alongside participants’ demographic and professional characteristics according to these groupings. As outlined in Table 5, the distribution of healthcare workers in the low, moderate and high perceived coercion groupings did not tend to differ significantly on most variables. The exceptions were (i) a larger number of men in the moderate perceived coercion (subscale) group, (ii) fewer participants with postgraduate degrees in the high scoring group, and (iii) fewer nurses in the low, and more medical doctors in the moderate groupings. It is noteworthy that of workplace factors, perceptions of feeling ‘supported by my team’ were significantly different between groups (F(2477) = 47.11, *p* < 0.001), and Bonferroni-corrected post hoc tests showed that each group was significantly different from the other on this measure (*ps* ≤ 0.002). Those in the low perceived coercion group rated support from the team as highest whereas those in the high perceived coercion group experienced least team support (Table 5).

**Table 5:** Participants’ demographic and workplace characteristics for the three perceived coercion groups (low, medium, high) in confirmatory factor analysis sample.

Variables	Low	Moderate	High	*p* value*
Perceived coercion subscale				
Internal Perceived Pressure, *M* (*SD*)	− 0.89 (1.21)	0.33 (1.30)	1.76 (1.02)	< 0.001
Perceived Coercion, *M* (*SD*)	− 0.38 (0.92)	1.68 (0.74)	1.85 (0.91)	< 0.001
External Perceived Pressure, *M* (*SD*)	− 2.01 (0.98)	− 1.51 (0.94)	1.10 (1.00)	< 0.001
Average Total Perceived Coercion, *M* (*SD*)	− 1.09 (0.67)	0.17 (0.58)	1.57 (0.61)	< 0.001
Demographics				
Age (years), *M* (*SD*)	42.1 (12.1)	39.2 (10.7)	39.8 (10.7)	0.053
Country of residence				
UK, n (%)	53 (27%)	71 (36%)	73 (37%)	0.158
Norwegian, n (%)	96 (34%)	111 (39%)	79 (28%)	0.068
Sex^a^				
Female, n (%)	107 (32%)	118 (35%)	109 (33%)	0.735
Male, n (%)	42 (29%)	63 (44%)	39 (26%)	0.028
Ethnicity^b^				
Caucasian, n (%)	130 (30%)	166 (39%)	134 (31%)	0.069
Non-Caucasian n (%)	19 (40%)	15 (31%)	14 (29%)	0.721
Relationship status				
Married/in a relationship, n (%)	108 (32%)	130 (38%)	101 (30%)	0.132
Not in a relationship, n (%)	40 (28%)	53 (37%)	52 (36%)	0.339
Postgraduate degree				
Yes, n (%)	84 (35%)	102 (42%)	57 (23%)	0.002
No, n (%)	66 (28%)	80 (33%)	93 (39%)	0.101
Workplace factors				
Role				
Nurse, n (%)	41 (23%)	68 (37%)	72 (40%)	0.009
Medical Doctor, n (%)	62 (33%)	85 (45%)	43 (22%)	0.001
Other, n (%)	46 (41%)	29 (26%)	37 (33%)	0.144
Years of Experience (years), *M* (*SD*)	17.0 (11.4)	14.7 (10.1)	14.5 (10.0)	0.060
Perception of support from team	6.20 (1.0)	5.66 (1.35)	4.64 (1.81)	< 0.001

Values are means (SD) or counts (%).

Low group: n = 149; moderate: n = 182; high: n = 152.

^a^ To avoid small cells, five participants were excluded from the sex count either because they did not disclose their sex/gender or disclosed ‘non-binary’.

^b^ Data missing for n = 5.

*p values relate to ANOVAs for age and years of experience, and χ^2^ goodness of fit tests for country, sex, ethnicity, relationship status, advanced degree status and professional role. Each level of the latter variables was tested separately.

#### Psychological distress, compassion-fatigue and coping in participants with low, moderate and high levels of perceived coercion

To explore the construct validity of the PPCS-HCW and its subscales, we examined their association with relevant psychological constructs (Table 6). In particular, low, moderate and high perceived coercion groups were compared on levels of distress, compassion fatigue and coping. We found that the groups differed on DASS-stress (F(2480) = 29.52, *p* < 0.001), DASS-anxiety ((F(2480) = 63.28, *p* < 0.001), and DASS-depression subscales (F(2480) = 42.87, *p* < 0.001; Table 6). Post-hoc tests showed that the differences in stress, anxiety and depression generally resided between low *versus* high and moderate* versus* high groups (Bonferroni corrected *p* values < 0.001; all low *v.* moderate comparisons: *p*s ≥ 0.190). Other indices of maladaptive psychological responses also differed between groups: compassion fatigue (F(2480) = 67.26, *p* < 0.001) and avoidant coping (F(2479) = 25.68, *p* < 0.001) but not approach coping (F(2480) = 1.38, *p* = 0.252). Again, post hoc tests showed that low *versus* high and moderate *versus* high differences were significant for avoidance coping (*p* values < 0.001), but not low *versus* moderate (*p* = 0.991). The three groups differed from each other on compassion fatigue (*p* values ≤ 0.048), although the difference between the high and the other two groups was clearly larger than low *versus* moderate comparison (Table 6). When perception of support from the team, previous experience of mental health condition and years of experience were controlled, none of the significant results changed (effect of perceived coercion group on distress, compassion fatigue and avoidant coping ANCOVAs: *p* values < 0.001; see Table S1 for comparative tests statistics with/without covariates).

**Table 6:** Distress severity (DASS-21 stress, anxiety, depression subscales), compassion fatigue and coping in the cluster analysis-defined groupings (low, moderate and high) of perceived coercion.

Variables	Low	Moderate	High	*p*value*
Psychological distress				
Stress	8.60 (8.05)	10.62 (9.89)	16.86 (11.11)	< 0.001
Anxiety	4.12 (5.21)	4.89 (5.91)	12.57 (10.06)	< 0.001
Depression	7.46 (8.88)	8.68 (9.03)	17.16 (12.00)	< 0.001
Other relevant psychological attributes				
Compassion fatigue	22.30 (7.54)	24.66 (8.94)	33.40 (9.79)	< 0.001
Avoidance coping	16.50 (4.40)	17.01 (4.21)	20.04 (5.41)	< 0.001
Approach coping	27.35 (6.84)	27.47 (6.75)	28.48 (6.05)	0.252

Values are *Means* (*SD*).

* = *p* values from one-way ANOVAs.

## Discussion

The present study investigated the concept of perceived coercion in HCW during the COVID-19 pandemic. To this end, we developed and validated a new scale for evaluating this concept in those with direct experience of working on the frontline during the pandemic. The novel scale—the PPCS-HCW—had good psychometric properties and its items were represented by three factors: perceived coercion per se, internal perceived pressures and external perceived pressures, which were confirmed in an independent sample using CFA. The three factors of the PPCS-HCW corresponded well to previously described sub-components of the perceived coercion construct^20^. This consistency demonstrates the robustness and adaptability of the concept across contexts (i.e., restrictive practice in mental health care vs. work responsibilities during a global pandemic; across countries) and target population (patients vs. healthcare staff). In addition, we found an association between perceived coercion and distress, compassion fatigue and avoidant coping.

Despite support for the construct of perceived coercion in its various forms, participants as a whole (phase 3 ‘confirmatory’ sample) did not show high overall levels of healthcare-related perceived coercion (average of total PPCS-HCW = 0.22 on a − 3 to + 3 scale). Nonetheless, this was reliably > 0. The clearest positive endorsement on items of the PPCS-HCW (i.e. highest average ‘agreement’, corresponding to higher levels of perceived coercion) related to autonomy, control and peer *expectations* on the perceived coercion subscale. It is noteworthy that the expectation items did not load on the external pressures subscale and suggest that expectations are qualitatively different to frank ‘peer pressure’. Indeed, there was general *disagreement* with items of the external pressures subscale (scores < 0).

The internal perceived pressures subscale scores were relatively low, but, on average > 0. Internal perceived pressure appeared to represent internal and ‘informal’ pressures based on internalised standards and expectations related to the healthcare worker role, including concerns about deviating from internalised professional expectations and standards. Notably, in the initial evaluation, the ‘*the public expected me to work with patients with COVID-19 in spite of the risk*’ item loaded on the *internal* (rather than external) perceived pressure factor which might indicate that, earlier in the pandemic, such expectations had been internalised (to a greater extent than expectations of professional colleagues). This item was later removed due to poor item-scale correlation in the confirmatory factor analysis; we interpret the reduction in correlation to potentially reflect public opinion having less direct impact on self-evaluations of being a ‘good’ healthcare worker over time.

The perceived coercion subscale represented experiences of (lack of) autonomy, control, and expectations of peers. The fact that the “peers/superiors expected me to work in spite of the risk” items loaded on this factor, suggests that a sense of autonomy and control can become closely intertwined with professional culture and the collective and dominant opinion in the workplace. The ‘external pressures’ factor was more clearly associated with frank compulsion from external ‘formal’ sources and consequences of not complying. It was noteworthy however that our participants, on average, disagreed with items in this factor (average scores < 0 on the − 3 to + 3 scale). However, according to the classification algorithm (k-means clustering) those in the ‘high’ group showed levels of perceived pressure that were 2.5–3.0 SD larger than the moderate or low perceived coercion groups.

Scores on other perceived coercion scales, such as the McArthur Perceived Coercion Scale, have typically been shown to be bimodally distributed, with a majority of participants scoring at one of the extremes (e.g.,^39^). The PPCS-HCW did not show obvious bimodality (see supplement). However, groups formed from cluster analysis showed that those with low and moderate versus high levels of perceived coercion differed in distress metrics and avoidance coping. As expected, those with high levels of overall perceived coercion were more distressed and used more avoidance coping. They also showed greater levels of compassion fatigue. These preliminary findings are consistent with the existing perceived coercion literature in mental health care, which shows a small direct effect of perceived coercion on psychological distress (e.g.,^26^). This suggests the relevance of perceived coercion in understanding contributors to poor mental health in healthcare workers during the pandemic.

The findings reported here have several theoretical-conceptual and clinical implications and suggest directions for further research. Although the PPCS-HCW was developed in the context of the COVID-19 pandemic, we believe that it is adaptable and could be used in healthcare research to investigate perceived coercion during other epidemic/pandemic events. The findings on the novel application of the concept of perceived coercion also provides a framework and vocabulary for clinicians, policy makers and other researchers to further investigate the experiences of autonomy and choice in deciding/advocating for own work responsibilities under circumstances which are potentially unsafe, where there is limited resources, significant uncertainty and/or multiple demands and stressors.

### Strengths and limitations

To our knowledge, the present study is the first to extend the concept of perceived coercion to the context of providing (rather than receiving) healthcare. Using a combination of inductive and deductive methods for item development, meant that we were able to account for first-hand experiences of perceived coercion during the early stages of the COVID-19 pandemic to develop a valid tool for assessing this construct. Psychometric equivalence was found across two European countries, despite differences in culture, healthcare resources and pandemic-specific pressures. Having a short context-specific measure of perceived coercion during healthcare emergencies can provide a helpful measure of the perceived coercion construct and its impact for healthcare workers.

A pragmatic approach was taken in the early stages of the item pool development due to the time-sensitive nature of the project. The relatively limited number of items in the initial item pool (i.e.,^15^) may have introduced a risk of missing important data or aspects of perceived coercion, such as items representing experiences of procedural justice (i.e., experiences of fairness and having a say in decision-making processes). Although procedural justice was not a commonly occurring theme in the initial item developing phase (i.e., interviews), the possible relevance of the concept became more apparent in the healthcare workforce over time. Including procedural justice items could have strengthened our understanding of the relevance of, for example, inclusive decision-making in contributing to (mitigating against) perceived coercion and its association with psychological distress. The significance of HCWs’ opportunity-cost (i.e., the time spent filling out surveys would mean less time taking care of patients or relaxing after a long day of work) upon participation was a decisive factor for our pragmatic approach. Although studies from previous health emergencies have shown that HCWs are willing to contribute to relevant research in hope of long-term benefits, reports of COVID-19 and research fatigue posed a challenge for research contributions. The need for a short and comprehensive survey served as our incentive to increase the likelihood of participation.

In addition to taking a pragmatic approach to pooling data, the context within which these data were pooled needs to be highlighted. For example, there were different policies in place for HCWs across countries and across time points, impacting on our ability to isolate the specific policies that contributed to higher level of perceived coercion. Moreover, our sampling methods may have biased our findings by attracting HCWs who had a certain perspective or interest in the study. In addition, the UK sample may have included participants from the wider SAFER study which itself sought to identify behaviours of HCWs which might have increased the risks of transmission. Thus, those who participated in the current study may have had a different perception of COVID-19-related work policies than those who had refused to take part or were subject to behaviour change interventions at later time points which could have altered their perspectives.

Generally, sample sizes were adequate for psychometric evaluation purposes. However, the current sample was represented by common characteristics of high-income countries, as the majority of participants were white, having completed higher levels of education, living in Europe, industrialised and democratic countries, and having a yearly household income of > £50,000. The generalisability of the present study to HCWs from minority backgrounds, lower socioeconomic status and non-democratic countries are therefore unclear. This is an important consideration given the evidence highlighting the disproportionate impact of the COVID-19 pandemic on HCWs from BAME and deprived backgrounds as well as from countries with varying degrees and duration of COVID-restrictions^56,57^.

### Directions for future research and conclusion

This project represented an opportunistic exploration of an understudied concept in the context of a global pandemic. The outcome of the project was an adaptable and easily administered scale assessing perceived coercion for frontline healthcare workers. Determining the predictive validity of the scale (e.g., in predicting long-term well-being or mental health) will require additional research. Future research using the PPCS-HCW, however, does not need to be restricted to infrequent global infectious disease events. More localised contagion episodes will also likely require healthcare workers to work under conditions where coercion may also be experience (i.e. perceived). The contextual differences between pandemic and epidemic events would likely require further validation of the PPCS-HCW in epidemic contexts. Future longitudinal studies would allow an examination of changes in perceived coercion to be examined over extended periods. Such repeated assessment (in large samples) would be especially helpful in determining the causal role of perceived coercion in psychological distress, compassion fatigue and maladaptive coping using causal modelling.

Although we hope the necessity for the use of PPCS-HCW in mass-contagion related research will be limited, it is unfortunately inevitable that future pandemics will occur and will require careful consideration of experiences of healthcare professionals that impact their mental health and general well-being, including the perception of coercion (mostly based on *internal* standards rather than external pressures).

## Data Availability

The datasets used and/or analysed during the current study available from the corresponding author on reasonable request.

## References

[1] Burki, T. Global shortage of personal protective equipment. *Lancet Infect Dis.* **20**, 785–786. 10.1016/s1473-3099(20)30501-6 (2020). 32592673 10.1016/S1473-3099(20)30501-6 PMC7314445

[2] Tabah, A. et al. Personal protective equipment and intensive care unit healthcare worker safety in the COVID-19 era (PPE-SAFE): An international survey. *J. Crit. Care* **59**, 70–75. 10.1016/j.jcrc.2020.06.005 (2020). 32570052 10.1016/j.jcrc.2020.06.005 PMC7293450

[3] Allan, S. M. et al. The prevalence of common and stress-related mental health disorders in healthcare workers based in pandemic-affected hospitals: A rapid systematic review and meta-analysis. *Eur. J. Psychotraumatol.* **11**, 1810903. 10.1080/20008198.2020.1810903 (2020). 33244359 10.1080/20008198.2020.1810903 PMC7678680

[4] Kisely, S. et al. Occurrence, prevention, and management of the psychological effects of emerging virus outbreaks on healthcare workers: rapid review and meta-analysis. *BMJ* **369**, m1642. 10.1136/bmj.m1642 (2020). 32371466 10.1136/bmj.m1642 PMC7199468

[5] Morgantini, L. A. et al. Factors contributing to healthcare professional burnout during the COVID-19 pandemic: A rapid turnaround global survey. *PLOS One* **15**, e0238217. 10.1371/journal.pone.0238217 (2020). 32881887 10.1371/journal.pone.0238217 PMC7470306

[6] Houlihan, C. F. et al. Pandemic peak SARS-CoV-2 infection and seroconversion rates in London frontline health-care workers. *Lancet* **396**, e6–e7. 10.1016/s0140-6736(20)31484-7 (2020). 32653078 10.1016/S0140-6736(20)31484-7 PMC7347344

[7] Zhu, J., Stone, T. & Petrini, M. The ethics of refusing to care for patients during the coronavirus pandemic: A Chinese perspective. *Nurs. Inq.* **28**, e12380. 10.1111/nin.12380 (2021). 32955787 10.1111/nin.12380 PMC7537035

[8] Lai, J. et al. Factors associated with mental health outcomes among health care workers exposed to Coronavirus Disease 2019. *JAMA Netw. Open* **3**, e203976–e203976. 10.1001/jamanetworkopen.2020.3976 (2020). 32202646 10.1001/jamanetworkopen.2020.3976 PMC7090843

[9] Cai, H. et al. Psychological impact and coping strategies of frontline medical staff in Hunan between January and March 2020 during the outbreak of Coronavirus disease 2019 (COVID-19) in Hubei, China. *Med. Sci. Monit.* **26**, e924171. 10.12659/msm.924171 (2020). 32291383 10.12659/MSM.924171 PMC7177038

[10] Chan, A. O. M. & Huak, C. Y. Psychological impact of the 2003 severe acute respiratory syndrome outbreak on health care workers in a medium size regional general hospital in Singapore. *Occupational Med.* **54**, 190–196. 10.1093/occmed/kqh027 (2004). 10.1093/occmed/kqh027 PMC7107861 15133143

[11] Chan, S. S. et al. The impact of work-related risk on nurses during the SARS outbreak in Hong Kong. *Fam. Commun. Health* **28**, 274–287. 10.1097/00003727-200507000-00008 (2005). 10.1097/00003727-200507000-00008 15958885

[12] Wu, P. et al. The psychological impact of the SARS epidemic on hospital employees in China: Exposure, risk perception, and altruistic acceptance of risk. *Can. J. Psychiatry* **54**, 302–311. 10.1177/070674370905400504 (2009). 19497162 10.1177/070674370905400504 PMC3780353

[13] Bai, Y. et al. Survey of stress reactions among health care workers involved with the SARS outbreak. *Psychiatric Serv.* **55**, 1055–1057. 10.1176/appi.ps.55.9.1055 (2004). 10.1176/appi.ps.55.9.1055 15345768

[14] Muller, A. E. et al. The mental health impact of the covid-19 pandemic on healthcare workers, and interventions to help them: A rapid systematic review. *Psychiatry Res.* **293**, 113441. 10.1016/j.psychres.2020.113441 (2020). 32898840 10.1016/j.psychres.2020.113441 PMC7462563

[15] Krishnamoorthy, Y., Nagarajan, R., Saya, G. K. & Menon, V. Prevalence of psychological morbidities among general population, healthcare workers and COVID-19 patients amidst the COVID-19 pandemic: A systematic review and meta-analysis. *Psychiatry Res.* **293**, 113382. 10.1016/j.psychres.2020.113382 (2020). 32829073 10.1016/j.psychres.2020.113382 PMC7417292

[16] Nguyen, L. H. et al. Risk of COVID-19 among front-line health-care workers and the general community: A prospective cohort study. *Lancet Public Health* **5**, e475–e483. 10.1016/S2468-2667(20)30164-X (2020). 32745512 10.1016/S2468-2667(20)30164-X PMC7491202

[17] Denning, M. et al. Determinants of burnout and other aspects of psychological well-being in healthcare workers during the Covid-19 pandemic: A multinational cross-sectional study. *PLOS One* **16**, e0238666. 10.1371/journal.pone.0238666 (2021). 33861739 10.1371/journal.pone.0238666 PMC8051812

[18] Greenberg, N., Docherty, M., Gnanapragasam, S. & Wessely, S. Managing mental health challenges faced by healthcare workers during covid-19 pandemic. *BMJ.* **368** (2020). 10.1136/bmj.m1211 32217624

[19] Lamb, D. et al. Psychosocial impact of the COVID-19 pandemic on 4378 UK healthcare workers and ancillary staff: Initial baseline data from a cohort study collected during the first wave of the pandemic. *Occupational Environ. Med.* **78**, 801–808 (2021). 10.1136/oemed-2020-107276 PMC8245285 34183447

[20] Szmukler, G. & Appelbaum, P. S. Treatment pressures, leverage, coercion, and compulsion in mental health care. *J. Mental Health* **17**, 233–244. 10.1080/09638230802052203 (2008).

[21] Klag, S., Creed, P. & O’Callaghan, F. Development and initial validation of an instrument to measure perceived coercion to enter treatment for substance abuse. *Psychol. Addict. Behav.* **20**, 463–470. 10.1037/0893-164x.20.4.463 (2006). 17176181 10.1037/0893-164X.20.4.463

[22] Steadman, H. J. & Redlich, A. D. A scale to measure perceived coercion in everyday life: A concept to inform research on the legal issues of coerced treatment. *Int. J. Forensic Mental Health* **5**, 167–171. 10.1080/14999013.2006.10471240 (2006).

[23] Hoge, S. K. et al. Patient, family, and staff perceptions of coercion in mental hospital admission: An exploratory study. *Behav. Sci. Law* **11**, 281–293. 10.1002/bsl.2370110306 (1993). 10150231 10.1002/bsl.2370110306

[24] Kinner, S. A. et al. Attitudes towards seclusion and restraint in mental health settings: Findings from a large, community-based survey of consumers, carers and mental health professionals. *Epidemiol. Psychiatr. Sci.* **26**, 535–544. 10.1017/s2045796016000585 (2017). 27515597 10.1017/S2045796016000585 PMC6998893

[25] Newton-Howes, G. & Mullen, R. Coercion in psychiatric care: Systematic review of correlates and themes. *Psychiatr. Serv.* **62**, 465–470. 10.1176/ps.62.5.pss6205_0465 (2011). 21532070 10.1176/ps.62.5.pss6205_0465

[26] Whitecross, F., Seeary, A. & Lee, S. Measuring the impacts of seclusion on psychiatry inpatients and the effectiveness of a pilot single-session post-seclusion counselling intervention. *Int. J. Ment. Health Nurs.* **22**, 512–521. 10.1111/inm.12023 (2013). 23682907 10.1111/inm.12023

[27] Link, B., Castille, D. M. & Stuber, J. Stigma and coercion in the context of outpatient treatment for people with mental illnesses. *Soc. Sci. Med.* **67**, 409–419. 10.1016/j.socscimed.2008.03.015 (2008). 18450350 10.1016/j.socscimed.2008.03.015

[28] RCN. *Clinical Guidance For Managing COVID-19.*, https://www.rcn.org.uk/covid-19 (2020).

[29] Wright, D., Peterson, W. & Gifford, W. *Nurses’ ethical considerations during a pandemic* (Canadian Nurses Association, 2020).

[30] Freer, J. Selfless sacrifice or failed by the state? Remembering nurses who have died from Covid-19. *Int. J. Nurs. Stud.* **113**, 103736 (2020). 32807561 10.1016/j.ijnurstu.2020.103736 PMC7391238

[31] Higgins, E. T. Self-discrepancy: A theory relating self and affect. *Psychol. Rev.* **94**, 319 (1987). 3615707

[32] Lidz, C. W., Mulvey, E. P., Arnold, R. P., Bennett, N. S. & Kirsch, B. L. Coercive interactions in a psychiatric emergency room. *Behav. Sci. Law* **11**, 269–280. 10.1002/bsl.2370110305 (1993).

[33] Lee, T. S. H., Tzeng, W. C. & Chiang, H. H. Impact of coping strategies on nurses’ well-being and practice. *J. Nurs. Scholarship* **51**, 195–204 (2019). 10.1111/jnu.12467 30806038

[34] Zhang, Y.-Y., Zhang, C., Han, X.-R., Li, W. & Wang, Y.-L. Determinants of compassion satisfaction, compassion fatigue and burn out in nursing: A correlative meta-analysis. *Medicine* **97**, e11086 (2018). 29952947 10.1097/MD.0000000000011086 PMC6242309

[35] Boateng, G. O., Neilands, T. B., Frongillo, E. A., Melgar-Quiñonez, H. R. & Young, S. L. Best practices for developing and validating scales for health, social, and behavioral research: A primer. *Front. Public Health* **6**, 149 (2018). 29942800 10.3389/fpubh.2018.00149 PMC6004510

[36] Qualtrics. *Qualtrics survey software*. Available at: https://www.qualtrics.com (2020).

[37] Object Planet. *Opinio survey software*. Available at: https://www.objectplanet.com/opinio/ (2020)

[38] Walker, N. F. et al. Detection of SARS-CoV-2 infection by saliva and nasopharyngeal sampling in frontline healthcare workers: An observational cohort study. *PLOS One* **18**, e0280908 (2023). 36706119 10.1371/journal.pone.0280908 PMC9882898

[39] Gardner, W. et al. Two scales for measuring patients’ perceptions for coercion during mental hospital admission. *Behav. Sci. Law* **11**, 307–321. 10.1002/bsl.2370110308 (1993). 10150233 10.1002/bsl.2370110308

[40] Maneesriwongul, W. & Dixon, J. K. Instrument translation process: A methods review. *J. Adv. Nurs.* **48**, 175–186 (2004). 15369498 10.1111/j.1365-2648.2004.03185.x

[41] Bandalos, D. *Measurement theory and applications for the social sciences*. (Guilford Publications, 2018).

[42] Patil, V. K., Singh, M. & Yadav, D. Parallel analysis in statistics: A review. *Journal of Statistical Computation and Simulation*, **87**(5), 953–966. (2017) 10.1080/00949655.2017.1293771.

[43] Thompson, B. & Daniel, L. G. Factor analytic evidence for the construct validity of scores: A historical overview and guidelines. *Educat. Psychol. Measurements* **52**, 197–208 (1996).

[44] Tabachnick, B. G., Fidell, L. S. & Ullman, J. B. *Using multivariate statistics* (Pearson, 2013).

[45] Field, A. *Discovering Statistics Using IBM SPSS Statistics* (SAGE Publications, 2013).

[46] Putnick, D. L. & Bornstein, M. H. Measurement invariance conventions and reporting: The state of the art and future directions for psychological research. *Developmental review.* **41**, 71–90 (2016). 27942093 10.1016/j.dr.2016.06.004 PMC5145197

[47] Byrne, B. M. *Structural equation modeling with AMOS: Basic concepts, applications and programming* 2nd edn. (Taylor & Francis, 2013).

[48] Kline, R. B. *Principles and practice of structural equation modeling* 4th edn. (Guilford Publications, 2015).

[49] McDonald, R. P. The Noncentrality Index for Factor Analysis: Theoretical and Practical Considerations. Multivariate Behavioral Research, **24**(3), 261–281. (1989) 10.1207/s15327906mbr2403_2.

[50] Ranieri, V. et al. COVID-19 wellbeing study: A protocol examining perceived coercion and psychological well-being during the COVID-19 pandemic by means of an online survey, asynchronous virtual focus groups and individual interviews. *BMJ Open* **11**, e043418. 10.1136/bmjopen-2020-043418 (2021). 33495259 10.1136/bmjopen-2020-043418 PMC7839305

[51] Lovibond, S. & Lovibond, P. F. The structure of negative emotional states: Comparison of the depression anxiety stress scales (DASS) with the beck depression and anxiety inventories. *Behav. Res. Therapy* **33**, 225–242 (1995). 10.1016/0005-7967(94)00075-u 7726811

[52] Heritage, B., Rees, C. S. & Hegney, D. G. The ProQOL-21: A revised version of the professional quality of life (ProQOL) scale based on Rasch analysis. *PLOS One* **13**, e0193478 (2018). 29489875 10.1371/journal.pone.0193478 PMC5831102

[53] Carver, C. S. You want to measure coping but your protocol’too long: Consider the brief cope. *Int. J. Behav. Med.* **4**, 92–100 (1997). 16250744 10.1207/s15327558ijbm0401_6

[54] Eisenberg, S. A., Shen, B.-J., Schwarz, E. R. & Mallon, S. Avoidant coping moderates the association between anxiety and patient-rated physical functioning in heart failure patients. *J. Behav. Med.* **35**, 253–261 (2012). 21660588 10.1007/s10865-011-9358-0

[55] Hutcheson, G. D. & Sofroniou, N. *The multivariate social Cientist: Introductory statistics using generalized linear models* (SAGE Publications Ltd, 1999).

[56] Kirby, T. Evidence mounts on the disproportionate effect of COVID-19 on ethnic minorities. *Lancet Respirat. Med.* **8**, 547–548 (2020). 32401711 10.1016/S2213-2600(20)30228-9 PMC7211498

[57] England, P. H. Disparities in the risk and outcomes of COVID-19. *Public Health England* (2020).

